# Patterns of high-flying insect abundance are shaped by landscape type and abiotic conditions

**DOI:** 10.1038/s41598-023-42212-z

**Published:** 2023-09-13

**Authors:** Eva Knop, Majken Leonie Grimm, Fränzi Korner-Nievergelt, Baptiste Schmid, Felix Liechti

**Affiliations:** 1 Department of Evolutionary Biology and Environmental Studies, University of Zurich, Reckenholzstrasse 191, 8046 Zürich, Switzerland; 2 Swiss Ornithological Institute, Sempach, Switzerland; 3 Agroecology and Environment, Agroscope, Reckenholzstrasse 191, 8046 Zurich, Switzerland; 4 Swiss Birdradar Solution, Winterthur, Switzerland

**Keywords:** Ecology, Agroecology, Animal migration, Biodiversity, Community ecology, Urban ecology

## Abstract

Insects are of increasing conservation concern as a severe decline of both biomass and biodiversity have been reported. At the same time, data on where and when they occur in the airspace is still sparse, and we currently do not know whether their density is linked to the type of landscape above which they occur. Here, we combined data of high-flying insect abundance from six locations across Switzerland representing rural, urban and mountainous landscapes, which was recorded using vertical-looking radar devices. We analysed the abundance of high-flying insects in relation to meteorological factors, daytime, and type of landscape. Air pressure was positively related to insect abundance, wind speed showed an optimum, and temperature and wind direction did not show a clear relationship. Mountainous landscapes showed a higher insect abundance than the other two landscape types. Insect abundance increased in the morning, decreased in the afternoon, had a peak after sunset, and then declined again, though the extent of this general pattern slightly differed between landscape types. We conclude that the abundance of high-flying insects is not only related to abiotic parameters, but also to the type of landscapes and its characteristics, which, on a long-term, should be taken into account for when designing conservation measures for insects.

## Introduction

Insects constitute a major part of the Earth’s biodiversity^1^ and provide many essential ecosystem services such as pollination or pest control^2–4^. Recent reports of a severe reduction of both insect abundance and diversity^5,6^, therefore, raised considerable concerns in the scientific community and public^7^, though also inconsistent patterns of changes in insect communities have been found^8^. So far, the change of landuse and climate have been identified as major driver of changes in insect abundance and diversity^9,10^, though we are only at the beginning of understanding it^6^. To our knowledge the current evidence on the occurrence and change of insect communities is based on insects recorded close to the ground and we know only little about high-flying insects. Also, we know little on how the suitability of the airspace as a habitat, i.e., how it differs between landscapes^11^, and accordingly how its suitability is affected by the current global changes, such as land-use changes or climate warming.

Research on high-flying insects lags behind that on high-flying birds^11^, most likely because they are more difficult to track. However, recent developments in radar technology allow now to continuously monitor flying insects over a wide range of altitudes^12,13^. Also, the algorithms to classify radar signals have improved^14–16^. Existing studies, however, have so far mostly focused on single sites (but see studies on the painted lady butterfly^17,18^) and did not yet account for variability among different landscape types, which would be needed for a better understanding of how global change affects high-flying insect communities.

Many insects use the airspace at high altitudes (up to several hundred meters^19^) for various important aspects of their life cycle, such as for mating (e.g., mayflies), migrating to new feeding sites (e.g., locusts or aphids), or seasonal migration (e.g., moths)^11^. In temperate regions, insect migration (i.e., a seasonal type of movement, that is repeated in time, space, and direction^20^) mainly occurs in spring and autumn, is directed towards higher latitudes, lower latitudes, respectively^21,22^, and can cause huge biomass fluxes^23^. In contrast, mating insects or insects migrating to new feeding sites seem to have their peak flying activity in summer with an increase in spring that takes place later for nocturnal than for diurnal insects^24^. Also, in summer high-flying insects were found to not fly in one specific direction^24^. However, we are only at the beginning of understanding those patterns.

To date, there are only few studies that investigated which abiotic factors influence the abundance of high-flying insects^11^. It seems that high-flying insects generally take advantage of the wind to travel, that their abundance depends on several meteorological parameters and varies over the course of 24 h^11,23^. For example, Hu et al.^23^ showed that while temperature and air pressure were positively related to high-flying insect abundance, wind speed and air humidity were negatively related. In spring and autumn, but not in summer, wind direction also had an effect. Chapman et al.^12^ found a daily pattern in insect abundances with an increase in the morning, a decrease in the afternoon and distinct peaks at dawn and dusk.

Similar to what is known from insects close to the ground^25–28^, the diversity and density of high-flying insects might differ between landscape types, such as between urbanized and rural areas. For example, it is well known, that the land-use intensity in a given landscape type, such intense agricultural practices in rural lowland areas have a negative effect on the diversity and abundance of insects e.g.,^29^. Also, even though insect diversity is known to decline with elevation due to harsher environmental conditions^30^, biodiversity (including insects) is currently found to be even higher in the mountains as compared to the lowlands, most likely due to the more pristine or less intense land-use there^31^. The idea, that the diversity and density of high-flying insects is related to the type of landscape, is, for example, also supported by a study on migratory hoverflies caught close to the ground: Compared to urbanised areas higher densities in lowland farmland areas were found during summer, whereas in spring (i.e. during migration), the migratory hoverflies were present earlier in urbanised areas, most likely as urbanised areas offered already flower resources at that time of the year^26^. Yet, we currently do not know whether also high-flying insect abundance differs between landscape types.

Here, we therefore asked, (i) how the abundance of high-flying insects is related to abiotic factors (temperature, wind and air pressure), (ii) how it differs between three different landscape types, namely urban, rural and mountainous areas, and (iii) how it changes over the course of 24 h depending on the landscape types, and. Similar to what has been found in previous studies^11,23^, we expected that temperature and air pressure have a positive effect while wind speed has a negative effect on the density of high-flying insects. Further, we expected no clear effect of wind direction as we focussed on high-flying insects during summer^23^. Based on studies focusing on insect abundances close to the ground, we expected the highest abundance in mountainous areas, followed by rural and urbanised areas. Furthermore, we expected that high-flying insect abundance changes over the course of 24 h with a peak around noon and less pronounced peaks in the morning and evening, and that this pattern is similar across landscape types. To answer these questions, we used data from vertical-looking radar devices, placed at six independent locations in Switzerland that differed in terms of landscape type.

## Methods

### Sampling design and data collection

**Figure 1:**
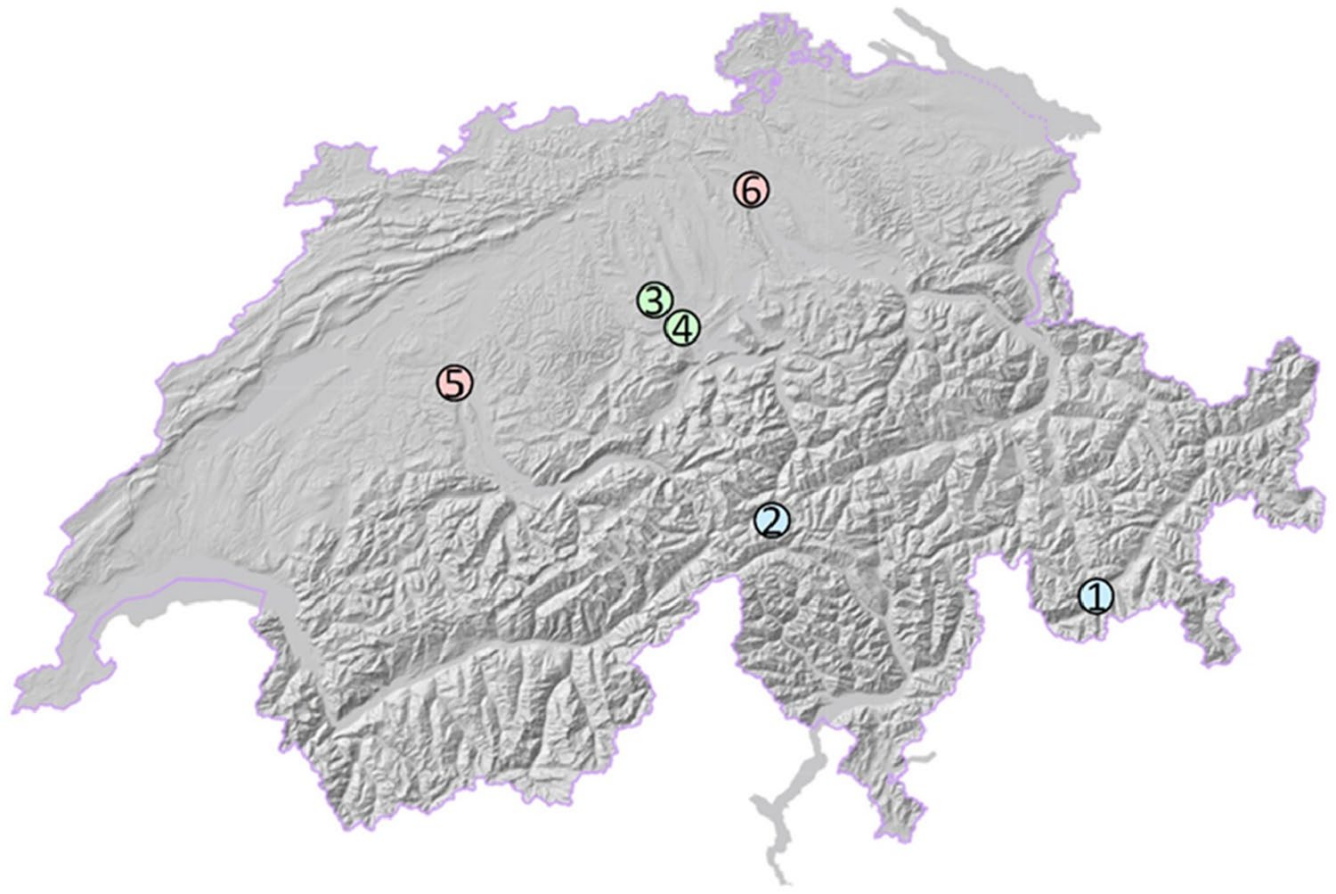
Map of the sites where radar data were collected. 1: Maloja; 2: Hospental; 3: Sempach; 4: Rothenburg; 5: Bern; 6: Zurich. Urban sites are indicated in light red, lowland rural sites in light green, mountainous sites in light blue. Hillshade computed from swissALTI3D, copyright swisstopo (www.swisstopo.admin.ch).

For tracking the abundance of high-flying insects, we used a total of four vertical-looking X-band radar devices of the type BirdScan MR1 (Swiss Birdradar Solution AG, www.swiss-birdradar.com, for technical details see Schmid et al.^15^). Data on high-flying insect activity was collected during up to six weeks in summer 2020 at six locations in Switzerland (Fig. 1, Supplementary Table 1). Three locations (Hospental, Maloja, Sempach) covered the whole period (9.7.2020–25.8.2020) whereas three covered two consecutive weeks each (Bern: 27.7.2020–11.8.2020; Rothenburg: 9.7.2020–27.7.2020; Zurich: 11.8.2020–25.8.2020; see also Supplementary Fig. 1). The locations differed in terms of type of landscapes: Two of them were in urban areas, i.e., within the boundaries of cities with more than 100,000 residents in the lowlands and more than 70% settlement area within a perimeter with a radius of 1 km (Supplementary Table 1). Four were in rural areas, in small towns or villages with less than 10,000 residents with less than 30% settlement area within a perimeter with a radius of 1 km, two of which were in the lowlands and the other two in mountainous terrain (Supplementary Table 1). The radius of 1 km was chosen for the perimeter defining a landscape sector because this is a size that is commonly chosen for studies on differences in insects between landscape types (e.g.,^26,28^). For more details regarding the land use within the perimeters see Supplementary Table 1.

The radar devices worked such that objects crossing the vertically emitted beam were automatically detected. The lowest height of detection was 40 m, and the maximum height of detection was dependent on the object’s size, with larger objects being detected farther up. Theoretically insects of 1cm diameter (e.g., *Syrphus ribesii*) could be detected up 350 m, with 2 cm (e.g., *Agrotis ypsilon*) up to 500 and with 5 cm (e.g., *Pantala flavescens*) up to 800 m. However, in practice, there are several factors affecting detection range. First, insect bodies are far from a sphere assumed for the theoretical calculations. Second, the position of an object within the beam is not known, thus the echo of large insects at the edge can be similar to small insects in the centre of the beam, and third, most insect sizes fall into the mie or even Raleigh range, which complicates the estimate of the insect size additionally. Based on field experiments with the release of insect specimens (unpublished), we assume that the detection range in this radar (BirdScan MR1) is about half of the theoretical calculations. Very small insects (e.g., aphids) cannot be detected as a single target: due to the wavelength (3 cm) the reflectivity of objects smaller than 0.5 cm drops exponentially^19^. Thus, a proper calibration of insect targets was not possible. Therefore, the densities are not absolute but relative measures, and since the radar systems are calibrated and identical, there is little difference in detection range between the devices. Insects were separated from other objects like birds and bats using an algorithm based on Zaugg et al.^16^ and Schmid et al.^15^. The operational modes of the radar devices were short pulse mode for 40 min per hour followed by 20 min in medium pulse mode. As longer pulses result in a lower resolution of the distance, only data from short pulse mode with a resolution of approximately 10 m were used for the analysis. Precipitation events and time frames with technical problems were excluded. From the filtered data, a traffic rate was calculated as a frequency-based measure for insect abundance. The measure corrects for the height dependent detection range and provides the number of insects crossing a theoretical cylinder of 1 km within an hour, see^15^. However, the resulting insect abundance represents a minimum abundance, which can only be compared between the identical radar systems.

### Abiotic variables

For explanatory variables, we procured weather data obtained from MeteoSwiss (www.meteoswiss.admin.ch). From the weather stations that were nearest to the radar sites (Supplementary Table 2), we used hourly data on air temperature at 2 m above the ground as well as air pressure at ground level. As we expected the wind to differ at the height the insects were flying from the wind at ground level, we extracted from the COSMO-1 model wind speed and mean wind direction at a height of 150 m above the ground at the respective radar locations. The COSMO-1 is a version of the COSMO (Consortium for small-scale modelling, www.cosmo-model.org) family of numerical weather prediction models with a grid box size of 1.1 km. The model is operated by MeteoSwiss (www.metoswiss.admin.ch). The height of 150 m corresponded to the rounded mean height of the insects detected by radar. The wind data were modelled for Maloja, Hospental and Sempach. Data from Sempach were also used as an approximation to the conditions in Rothenburg, Bern and Zurich, as these locations all lie in the Swiss plateau within less than 60 km distance.

### Statistical analyses

The number of insects per hour (traffic rate) was analysed using a linear mixed model assuming Gaussian distribution of the residuals. To meet that assumption the number of insects was square-root transformed. We included an autocorrelation structure of order 1 for the residuals. The order of the temporal autocorrelation was found by inspecting the partial autocorrelations using the acf function^32^. To answer our questions we included location nested in landscape type, first and second order orthogonal polynomials of air temperature and wind speed, wind direction, air pressure, hour of the day, and the interaction hour of the day with landscape type as predictors. To account for the nested structure of location (six levels: Maloja, Hospental, Sempach, Rothenburg, Zurich, Bern) in landscape type we used location as predictor and defined the first two contrasts to measure differences between landscape types (mountainous vs. rural, and mountainous vs. urban respectively). The last three contrasts measured the difference between the two locations within each landscape type. Hour of the day was included as a factor with 24 levels (0 am, 1 am, 2 am, 3 am, 4 am, 5 am, 6 am, 7 am, 9 am, 10 am, 11 am, 12 am, 13 pm, 14 pm, 15 pm, 16 pm, 17 pm, 18 pm, 19 pm, 20 pm, 21 pm, 22 pm, 23 pm), and wind direction had four levels (south (121°–210°), west (211°–300°), north (301°–30°), east (31°–120°). Air pressure, temperature, and wind speed were centred and scaled. We fitted the model using Hamiltonian Monte Carlo as implemented in Stan^33^, and we accessed Stan via the R-interface brms^34^. We used weakly informative prior distributions for the model parameters, specifically a normal distribution with mean of zero and a standard deviation of 30. We simulated four chains of length 2000 each and used the last 1000 of each chain for describing the posterior distributions of the model parameters. Convergence of the chains were assessed by the standard diagnostic plots and statistics^35^. To assess model fit, we compared the distribution (histograms), means, standard deviation and range of the posterior predictive distribution with the data (posterior predictive model checking). We report the mean of the posterior distribution as point estimates and the 2.5% and 97.5% quantiles of the posterior distributions as lower and upper limits of the 95% credible intervals (CrI)^36^. All analyses were done in R version 4.0.2 (R core team 2020).

## Results

**Figure 2:**
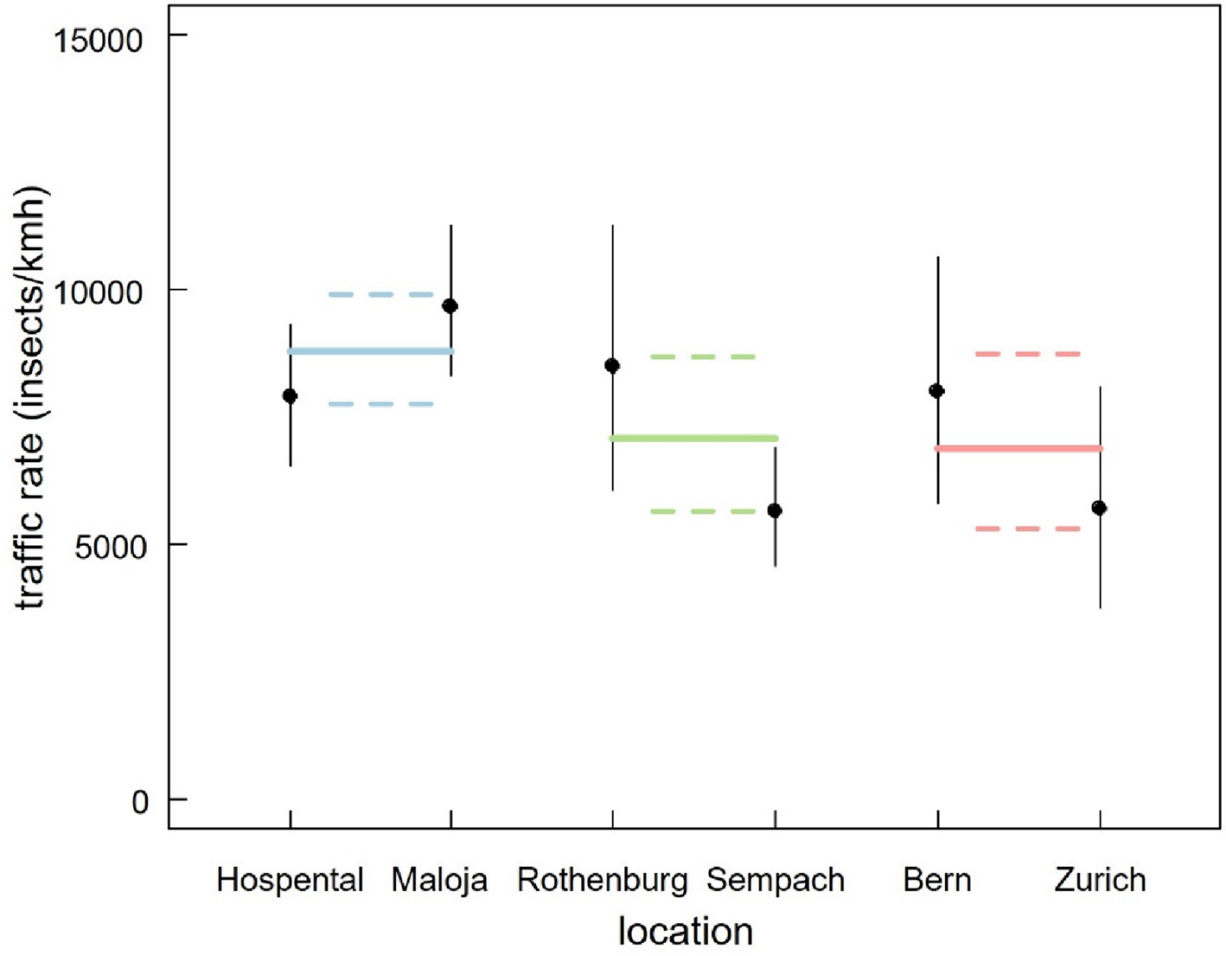
Mean hourly traffic rates at the 6 locations (Hospental: N = 1104, Maloja: N = 1110, Rothenburg: N = 295, Sempach: N = 1099, Bern: N = 316, Zurich: N = 319), grouped according to the three different landscape types. Estimates and 95% credible intervals are given. Coloured lines show the estimates and 95% credible intervals of the average per landscape type (light blue: mountainous, light green: rural lowlands, light red: urban lowlands).

**Figure 3:**
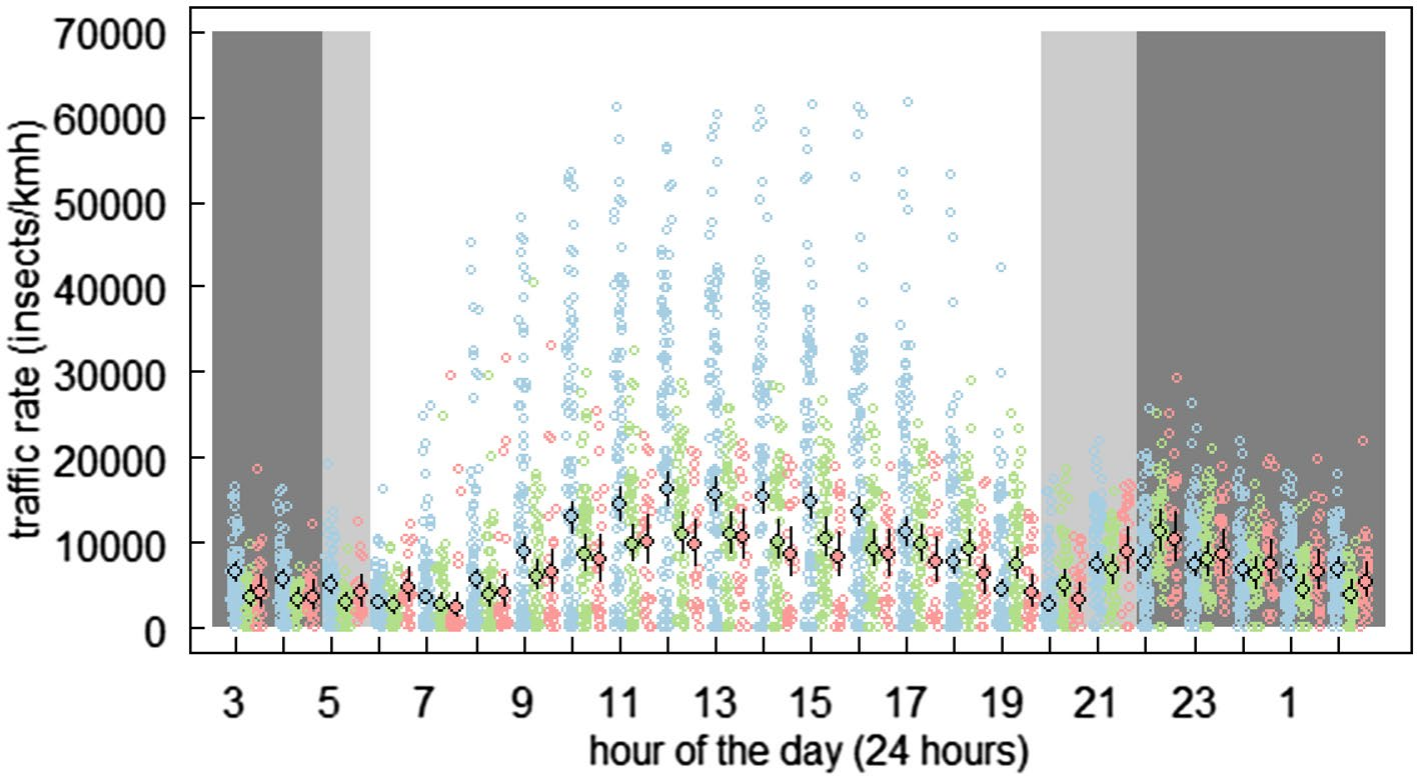
Hourly traffic rates over the course of 24 h and for the three landscape types (blue: mountainous, green: rural lowland, red: urban lowland). Estimates and 95% credible intervals are given with filled points, black lines respectively. Open points show the raw data. Grey areas indicate the period between dusk and dawn. As the experiment run over six weeks the time of dusk and dawn shifted. Accordingly, the range of dusk and dawn is indicated by light grey areas. Time is given as MESZ.

The average hourly traffic rate differed between the different landscape types (Fig. 2, Supplementary Table 4): Compared to mountainous landscapes the overall traffic rate was in urban landscapes lower (mean difference (lower and upper limits of the 95% credible interval) = − 1914 (− 3745, 99), Fig. 2). Similarly, rural landscapes had a lower traffic rate compared to mountainous landscapes (− 1709 (− 3472, 128), Fig. 2). The higher traffic rate in mountainous landscapes compared to rural and urban landscapes was more pronounced when accounting for the hourly differences over the course of 24 h: Generally, there was an increase in traffic rates in the morning, a decrease in the afternoon and a peak after sunset (Fig. 3, Supplementary Table 4). This overall pattern was similar in the three landscape types. However, in mountainous landscapes the hourly traffic rates around noon were higher compared to rural and urban landscapes (Fig. 3, Supplementary Table 4). Interestingly, after sunset and during the night the difference in traffic rates between the three landscape types disappeared. Further, after sunset, there was a peak in traffic rates in urban and rural sites, which was similar high to the peak around noon (Fig. 3, Supplementary Table 4). In contrast, in mountainous landscapes the peak after sunset was lower than the daytime peak around noon (Fig. 3, Supplementary Table 4). Overall, the daily traffic rate declined over the course of the three two-week sampling periods (Supplementary Fig. 1). Further, the height distribution of the detected insects was similar between the locations, across the three two-week sampling periods, but it was higher during the night than day (Supplementary Fig. 2).

**Figure 4:**
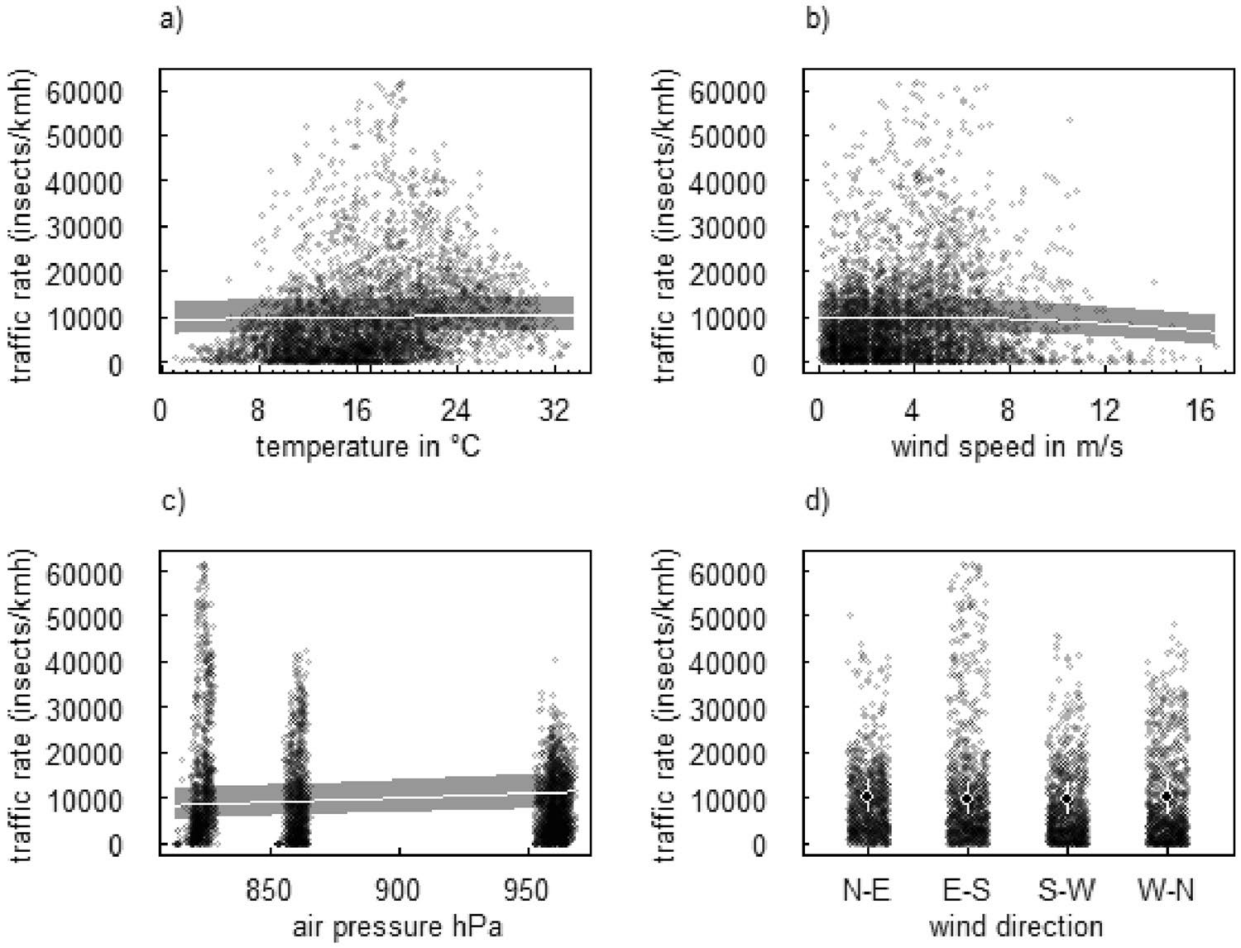
Relationship and 95% credible intervals between insect traffic rate and temperature (**a**), wind speed (**b**), air pressure (**c**), and wind direction (**d**). Wind direction indicates the direction from which the wind was coming, namely from the North to East (N–E), East to South (E–S), South to West (S–W), and West to North (W–N). Transparent points show the raw data.

The hourly traffic rate positively correlated with air pressure and showed a non-linear relationship with wind speed in which traffic rate peaked between 2 and 5 m/s and decreased with higher speeds (Fig. 4, Supplementary Table 4). There was, however, no clear relationship between hourly traffic rate and temperature and wind directions (Fig. 4, Supplementary Table 4). As the lack of a relationship between hourly traffic rate and temperature was unexpected, we also plotted the raw data of the relationship for the different landscape sectors separately (Supplementary Fig. 4), and for night- and daytime, respectively (Supplementary Fig. 5). Slightly positive correlations between temperature and hourly traffic rate can be seen at all study sites. However, these correlations seem to be explained by other variables such as time of the day, air pressure and wind speed in our multi-variate model.

## Discussion

Abundances of high-flying insects tended to differ between the landscapes, with the highest density of high-flying insects in mountain areas. Thus, it seems that in addition to the abiotic conditions, which have previously been found to be related to the density of high-flying insects^11,21,37^, factors related to the landscape type are also related to it, which was expected but has to our knowledge not yet been reported so far. Even though several parameters differ between mountainous areas and rural areas in the plateau, the poor state of biodiversity due to intense agricultural practices including the use of pesticides in the lowland might be a reason for the low abundances of high-flying insects in rural areas, similar to as it has been found in studies on insects close to the ground e.g.,^29^. A previous study conducted in Switzerland found that insects, especially at night, showed a more directed flight behaviour already as from mid-July onwards^24^. We therefore cannot rule out that the high density in mountainous areas was partly also due to topography, i.e., a concentration of long-range insect movements along valleys to avoid high mountain crossings. On the other hand densities of high-flying insects were only during daytime higher in the mountainous landscapes, which questions the latter idea. Further studies are needed to disentangle the role of the different landscape components (such as land use or topography) in influencing the density of high-flying insects.

Based on previous findings for migrating hoverflies close to the ground^26^, we expected more high-flying insects in rural landscapes compared to urban landscapes due to the larger area of vegetated areas, which offer more habitats for insects. We further expected the effect to be even more pronounced at night due to the negative effect of artificial light at night on insect abundances in urban areas^38–40^. Unlike our expectation, the difference between rural and mountainous landscapes was similar to the difference between urban and mountainous landscapes. As we do not know the identity of the species, it is possible that the relatively high density of high-flying insects in urban areas after sunset originated from species adapted to the urban environment. For insects active during daytime, at least, it is known, that there are many urban exploiters, i.e., that some insects perform very well in cities e.g.,^25,27,28^. Alternatively, the relative high abundance of high-flying insects in cities despite unfavourable habitat conditions could be due to long-distance attraction to artificial light at night as it is for example known for birds^41^. This idea, however, requires further investigations as to the best of our knowledge, to date not much is known about long-distance attraction of insects to urban areas. Experimental studies on insect attraction to light concluded that they are attracted to artificial light sources from distances below 100 m^39^. However, those studies focused on a very limited set of species and used very discrete light sources, such as streetlamps. The large-scale light pollution in form of skyglow produced by a city is different, and it thus should receive more attention in near future.

Similar to a previous study by Chapman et al.^12^ we found daily peaks of high-flying insects around midday and after sunset. However, in contrast to their study, we found no peak of high-flying insect abundance at dawn. One explanation for this finding could be, that the species composition of the communities assessed in the two studies differed: Studies conducted close to the ground have shown that species groups have different activity peaks over the course of 24 h^42^. Thus, the peaks of high-flying insect abundance probably reflected the activity patterns of distinct species groups. The latter could potentially also explain why in Chapman et al.^12^ found a changing peak intensity over the course of three months during which the species composition also very likely has changed. Interestingly, the circadian patterns of insect activity over 24 h were different for high-flying insects and flower-visiting insects close to the ground^42^. Close to the ground the activity peak of flower-visiting insects is clearly around noon. One explanation might be that the airspace and habitats close to the ground are used for different activities, which might be best performed at different times. For example, visiting flowers also involves the visual attraction of insects and therefore probably has its peak during daytime, though the pattern might vary, depending on abiotic conditions, i.e., under high solar radiation^43^. We are, however, only at the beginning of understanding which species are recorded and thus explanations currently remain speculative.

The positive relationship of high-flying insect densities with air pressure, the non-linear relationship with wind speed, respectively, is in line with previous findings^11,21,23^ and might be explained by their influence in the insect’s capacity of flight. For example, the non-linear relationship of high-flying insect densities with wind speed might be explained by the fact that strong winds are known to reduce the insect’s control over the flight direction on the one hand. On the other hand, slow winds are known to less support the insects with the consequence that movements cost more energy or that insects reach less far^19^. In contrast to previous studies, we did not find a clear relationship between temperature and abundance of high-flying insects, which is surprising as low temperatures make it difficult for an insect to move^19^. A reason for this unexpected finding might be, that during the summer months temperature might be less limiting than early or late in the season. Alternatively, in our model, part of the variation caused by temperature might already be explained by taking the hour as a predictor in our model. The latter, however, is also unlikely to completely explain the lack of a clear relationship with temperature in our multi-factorial model, as when removing hour from the model the results did not change with respect to the temperature effect (results not shown). We must also consider that the temperature height profiles may differ between sites, but we only had access to temperature 2 m above the ground. Thus, our results were indeed most likely due to ground temperature not being a main factor during our sampling period. Further studies are needed to find out under which conditions temperature is limiting for the density of high flying insects. In line with previous studies on high-flying insect abundances in summer^23^, we also did not find an effect of the wind direction. This is in contrast to what is known for migratory insects^23^, and to what is reflected by the proportion of directed flights in summer, which is rather low compared to autumn^24^.

As discussed above, insect traffic rates differed between landscape types, depended on abiotic conditions, and varied over the course of 24 h. Due to methodological limitations (farther up detection of larger insects), the differences could theoretically also have been caused by differences in body size distribution of the insect communities or differences in the height at which the insects flew. However, comparing height distributions between sites (Supplementary Fig. 2) did not support these assumptions. Even though we cannot fully exclude those two mechanisms, we do not believe they were dominating the pattern. A systematic change of the size distribution of the insects within a community due to the investigated factors is, to our knowledge, not known from studies at that scale close to the ground. For example, a more extensive management of wheat fields might locally shift the mean body size distribution of insects within that field^44^, but at the scale of entire landscapes, we are not aware of a study suggesting a systematic body size distribution of a community. As for the altitudinal flight pattern of insects, the most likely factor that influenced it were the abiotic conditions as they directly impacted the insect’s ability to fly, as discussed above. However, abiotic conditions vary over much larger distances and should be much more dependent on general weather conditions than on landscape types, for example. As previous studies on tracking insects with radars e.g.,^21,23,24^, we thus are confident that potential differences in insect composition did not have a major impact on our measurements of insect abundance.

Overall, we thus conclude that the abundance of high-flying insects is not only related to abiotic parameters, but also to the type of landscape. Pronounced differences between landscape type but also between the two sites within the landscape types show that the habitat on the ground influences the abundance of high-flying insects. Further studies should aim at disentangling the relationship of the different characteristics of landscapes, such as topography and land use, with the density of high-flying insects. On a long-term, this will help to identify the specific habitat characteristics needed for promoting high-flying insects, a basic knowledge needed for designing effective conservation measures for insects.

### Supplementary Information

Supplementary Information.

## Data Availability

The data analysed in this study is available in the Zenodo repository, https://doi.org/10.5281/zenodo.7014700.
