# Supplementary material for: Patterns of high-flying insect abundance are shaped by landscape type and abiotic conditions
Source: Sci Rep. 2023 Sep 13;13:15114. doi: 10.1038/s41598-023-42212-z (PMC10499926; doi:10.1038/s41598-023-42212-z)
Supplement: Supplementary file 1 — Supplementary Information. [file 41598_2023_42212_MOESM1_ESM.docx]

**Supplementary table 1.** Places and times radar data was collected. Altitudes are given in meters above the sea (m.a.s.l.). The coordinates correspond to the World Geodetic System 84 (WGS84). The landuse within a perimeter of a radius of 1 km was obtained from [www.swisstopo.ch](http://www.swisstopo.ch).

| **Location** | **Coordinates** | **Altitude (m.a.s.l.)** | **Landscape type** | **Land use (radius 1 km)** | **Date** |
| --- | --- | --- | --- | --- | --- |
| Maloja | 46.4055 N/ 9.7023 E | 1809 | rural, mountainous | agriculture (39%) forest (36%) settlement (8%) rest (17%) | 9.7.2020 -25.8.2020 |
| Hospental | 46.6212 N/ 8.5695 E | 1493 | rural, mountainous | agriculture (65%) forest (27%) settlement (5%) rest (3%) | 9.7.2020 -25.8.2020 |
| Sempach | 47.1283 N / 8.1923 E | 511 | rural, plateau | agriculture (34%) forest (1%) settlement (30%) rest (lake) (35%) | 9.7.2020 -25.8.2020 |
| Rothenburg | 47.0834 N /  8.2440 E | 487 | rural, plateau | agriculture (68%) forest (13%) settlement (18%)  rest (1%) | 9.7.2020 -27.7.2020 |
| Bern | 46.9490 N /  7.4803 E | 542. | urban, plateau | agriculture (17%) forest (9%) settlement (74%) rest (0%) | 27.7.2020 -11.8.2020 |
| Zurich | 47.3932 N /  8.5257 E | 408 | urban, plateau | agriculture (0%) forest (7%) settlement (89%)  rest (4%) | 11.8.2020 -25.8.2020 |

**Supplementary table 2.** Locations of the weather stations where temperature and air pressure were measured. The coordinates correspond to the World Geodetic System 84 (WGS84).

| **Location** | **Coordinates** | **Associated radar site** |
| --- | --- | --- |
| Segl-Maria | 46.432331 N / 9.762325 E | Maloja |
| Andermatt | 46.630914 N / 8.580553 E | Hospental |
| Egolzwil | 47.179428 N / 8.004758 E | Sempach, Rothenburg |
| Lucerne | 47.036439 N / 8.301022 E | Sempach, Rothenburg |
| Mosen | 47.243842 N / 8.232828 E | Sempach, Rothenburg |
| Bern/Zollikofen | 46.990744 N / 7.464061 E | Bern |
| Koppigen | 47.11885 N / 7.605503 E | Bern |
| Zurich Affoltern | 47.427694 N / 8.517953 E | Zurich |
| Zurich Fluntern | 47.377925 N / 8.565742 E | Zurich |

**Supplementary table 3.** Ranges (min, max) and means of measured weather data by location. Where several weather stations were at a similar distance to a radar site, we took the mean of the values (see Supplementary table 2 for the location of the weather stations). The wind speed was extracted from the COSMO-1 model at a height of 150 m above ground at the respective radar locations. The COSMO-1 is a version of the COSMO (Consortium for small-scale modelling, [www.cosmo-model.org](http://www.cosmo-model.org)) family of numerical weather prediction models with a grid box size of 1.1 km, and it is operated by MeteoSwiss ([www.metoswiss.admin.ch](http://www.metoswiss.admin.ch)).

| **Location** | **Temperature (°C)** | **Wind speed (m/s)** | **Air pressure (hPa)** |
| --- | --- | --- | --- |
| Maloja | 2.4-23.7, ø 12.6 | 0.1-16.7, ø 4.3 | 813.2-829.4, ø 823.0 |
| Hospental | 1.2-25.8, ø 13.9 | 0.1-11.4, ø 4.2 | 852.5-865.3, ø 860.1 |
| Sempach | 10.1-33.2, ø 19.9 | 0.0-14.8, ø 3.0 | 955.4-968.2, ø 961.8 |
| Rothenburg | 10.7-28.5, ø 18.9 | 0.1-11.6, ø 3.1 | 957.7-968.2, ø 962.7 |
| Bern | 8.8-33.6, ø 20.7 | 0.0-14.8, ø 2.9 | 952.8-961.8, ø 957.4 |
| Zurich | 11.4-32.6, ø 21.2 | 0.2-14.4, ø 3.0 | 952.7-963.0, ø 957.6 |

**Supplementary table 4.** Model parameters and 95% credible intervals of the model analysing the hourly traffic rates (mountainous landscapes: N=2214, rural landscapes: N=1394, urban landscape: N=635). Temperature and wind speed are included as first (temperature 1, wind speed 1) and second (temperature 2, wind speed 2) order orthogonal polynomials. Intercept: wind direction (North to East), 3-4 am MESZ, mountainous landscape.

|  | **Estimate** | **Est.Error** | **lower 95% CI** | **upper 95% CI** |
| --- | --- | --- | --- | --- |
| intercept | 180.14 | 34.65 | 113.74 | 248.42 |
| temperature 1 | 50.9 | 30.37 | -7.99 | 111.93 |
| temperature 2 | -9.22 | 27.7 | -63.05 | 43.77 |
| wind speed 1 | -43.58 | 22.38 | -87.94 | 0.59 |
| wind speed 2 | -72.51 | 19.5 | -110.28 | -34.49 |
| air pressure | 6 | 1.86 | 2.4 | 9.61 |
| wind direction E-S | -2.22 | 1.19 | -4.51 | 0.1 |
| wind direction S-W | -2.2 | 1.15 | -4.44 | 0.09 |
| wind direction W-N | -0.26 | 1.19 | -2.62 | 2.07 |
| day of the year | -0.47 | 0.16 | -0.79 | -0.17 |
| 4-5 am | -5.48 | 2.43 | -10.26 | -0.71 |
| 5-6 am | -8.58 | 3.14 | -14.76 | -2.52 |
| 6-7 am | -25.12 | 3.58 | -32.07 | -18.32 |
| 7-8 am | -21.13 | 3.93 | -28.89 | -13.62 |
| 8-9 am | -5.1 | 4.18 | -13.27 | 2.97 |
| 9-10 am | 14.98 | 4.28 | 6.49 | 22.98 |
| 10-11 am | 33.68 | 4.35 | 25.29 | 41.88 |
| 11-12 am | 40.55 | 4.44 | 31.83 | 49.23 |
| 12-13 pm | 47.32 | 4.51 | 38.48 | 56.26 |
| 13-14 pm | 45.03 | 4.53 | 36.08 | 53.67 |
| 14-15 pm | 43.5 | 4.49 | 34.61 | 52.19 |
| 15-16 pm | 41.19 | 4.52 | 32.37 | 50.26 |
| 16-17 pm | 36.24 | 4.57 | 27.32 | 45.09 |
| 17-18 pm | 25.41 | 4.62 | 16.22 | 34.29 |
| 18-19 pm | 7.57 | 4.58 | -1.49 | 16.47 |
| 19-20 pm | -12.64 | 4.46 | -21.39 | -4.16 |
| 20-21 pm | -26.84 | 4.32 | -35.39 | -18.4 |
| 21-22 pm | 6.41 | 4.2 | -2.09 | 14.4 |
| 22-23 pm | 8.12 | 4.04 | 0.25 | 16.09 |
| 23-24 pm | 5.61 | 3.85 | -2.04 | 13.14 |
| 0-1 am | 1.75 | 3.55 | -5.26 | 8.65 |
| 1-2 am | 0.67 | 3.13 | -5.57 | 6.7 |
| 2-3 am | 2.65 | 2.39 | -2.05 | 7.4 |
| contrast mountain-rural | -8.77 | 7.55 | -24.17 | 5.79 |
| contrast mountain-urban | -9.42 | 8.12 | -25.43 | 6.27 |
| contrast mountain-mountain | 9.92 | 5.37 | -0.77 | 20.37 |
| contrast rural-rural | -17.35 | 8.42 | -33.65 | -1.15 |
| contrast urban-urban | -14.26 | 9.88 | -33.59 | 4.93 |
| 3-4 am : landscape urban | -6.11 | 8.38 | -23.13 | 10.48 |
| 4-5 am : landscape urban | -5.52 | 8.46 | -21.87 | 11.6 |
| 5-6 am : landscape urban | 2.14 | 8.38 | -14.76 | 18.43 |
| 6-7 am : landscape urban | 23.31 | 8.48 | 6.43 | 39.89 |
| 7-8 am : landscape urban | -0.39 | 8.37 | -17.24 | 16.02 |
| 8-9 am : landscape urban | -1.85 | 8.46 | -18.39 | 14.98 |
| 9-10 am : landscape urban | -5.44 | 8.51 | -22.02 | 11.29 |
| 10-11 am : landscape urban | -14.81 | 8.48 | -31.35 | 1.73 |
| 11-12 am : landscape urban | -10.37 | 8.31 | -26.21 | 5.93 |
| 12-13 pm : landscape urban | -19.71 | 8.3 | -35.73 | -3.56 |
| 13-14 pm : landscape urban | -12.56 | 8.45 | -28.83 | 4.35 |
| 14-15 pm : landscape urban | -21.22 | 8.64 | -37.74 | -4.24 |
| 15-16 pm : landscape urban | -20.37 | 8.5 | -36.97 | -3.8 |
| 16-17 pm : landscape urban | -14.55 | 8.63 | -31.53 | 2.27 |
| 17-18 pm : landscape urban | -7.6 | 8.58 | -24.16 | 9.01 |
| 18-19 pm : landscape urban | 0.08 | 8.45 | -16.58 | 16.36 |
| 19-20 pm : landscape urban | 6.34 | 8.48 | -10.53 | 22.43 |
| 20-21 pm : landscape urban | 14.03 | 8.34 | -3.01 | 30.13 |
| 21-22 pm : landscape urban | 17.68 | 8.36 | 1.13 | 34.02 |
| 22-23 pm : landscape urban | 23.18 | 8.37 | 7.13 | 39.35 |
| 23-24 pm : landscape urban | 16.37 | 8.5 | 0.08 | 33.35 |
| 0-1 am : landscape urban | 14.55 | 8.5 | -1.88 | 31.53 |
| 1-2 am : landscape urban | 10.09 | 8.42 | -6.3 | 26.28 |
| 2-3 am : landscape urban | 0.31 | 8.3 | -15.9 | 16.44 |
| 3-4 am : landscape rural | -11.53 | 7.33 | -25.82 | 3.06 |
| 4-5 am : landscape rural | -7.41 | 7.44 | -22.11 | 7.46 |
| 5-6 am : landscape rural | -8.65 | 7.37 | -22.45 | 6.29 |
| 6-7 am : landscape rural | 4.27 | 7.48 | -10.13 | 19.64 |
| 7-8 am : landscape rural | 2.89 | 7.51 | -11.56 | 18.02 |
| 8-9 am : landscape rural | -4.66 | 7.5 | -19.07 | 10.15 |
| 9-10 am : landscape rural | -9.06 | 7.44 | -23.41 | 5.51 |
| 10-11 am : landscape rural | -12.26 | 7.43 | -26.81 | 2.55 |
| 11-12 am : landscape rural | -12.56 | 7.36 | -26.88 | 2.05 |
| 12-13 pm : landscape rural | -13.69 | 7.33 | -27.69 | 0.72 |
| 13-14 pm : landscape rural | -11.93 | 7.35 | -26.52 | 2.42 |
| 14-15 pm : landscape rural | -13.98 | 7.33 | -28.12 | 0.3 |
| 15-16 pm : landscape rural | -10.04 | 7.35 | -24.6 | 4.49 |
| 16-17 pm : landscape rural | -11.9 | 7.44 | -26.4 | 2.5 |
| 17-18 pm : landscape rural | 2.02 | 7.51 | -12.16 | 16.96 |
| 18-19 pm : landscape rural | 16.96 | 7.57 | 2.66 | 32.46 |
| 19-20 pm : landscape rural | 27.62 | 7.48 | 13.24 | 42.35 |
| 20-21 pm : landscape rural | 27.55 | 7.44 | 13.46 | 42.14 |
| 21-22 pm : landscape rural | 4.12 | 7.45 | -9.99 | 19.28 |
| 22-23 pm : landscape rural | 27.12 | 7.45 | 12.5 | 42.05 |
| 23-24 pm : landscape rural | 11.91 | 7.44 | -3.08 | 26.39 |
| 0-1 am : landscape rural | 5.96 | 7.43 | -8.74 | 20.77 |
| 1-2 am : landscape rural | -4.62 | 7.42 | -18.91 | 10.23 |
| 2-3 am : landscape rural | -10.66 | 7.48 | -25.17 | 4.47 |


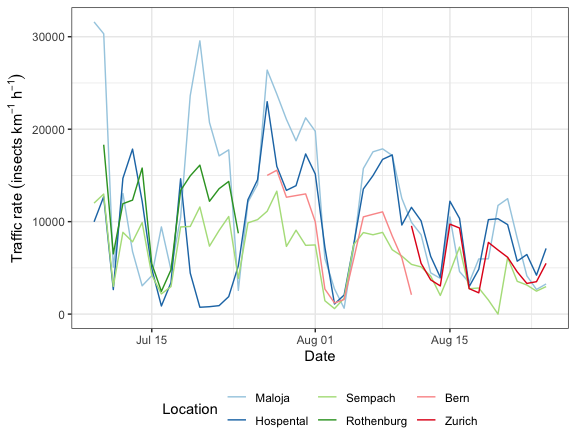


**Supplementary figure 1.** Daily mean traffic rate at the different locations. Mountainous landscapes (Maloja and Hospental) are depicted in blue colours, rural landscapes (Sempach and Rothenburg) in the lowlands in green colours, urban landscapes (Bern and Zurich) in red colours.


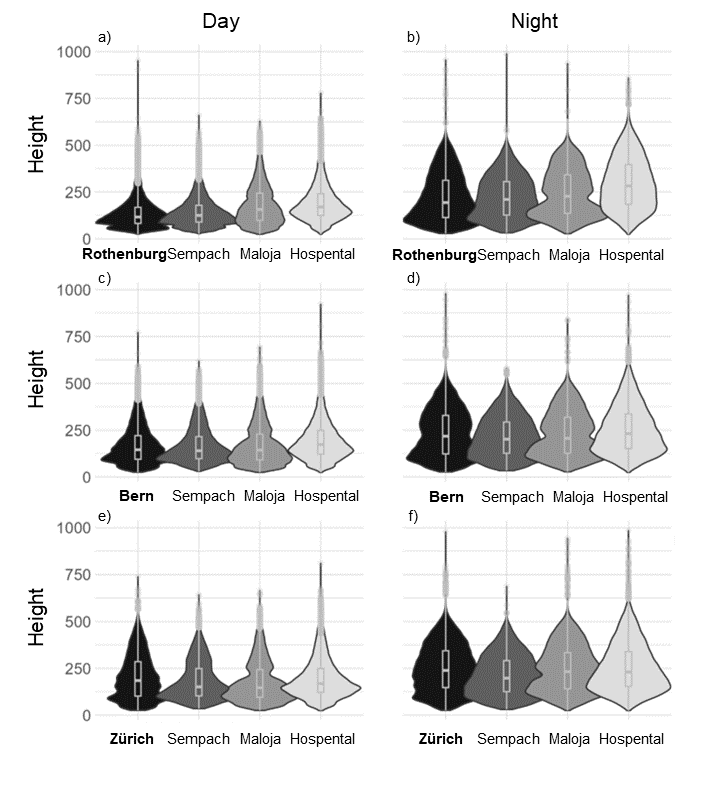


**Supplementary figure 2.** Height distribution of the detected individual insects, separate for the different locations, the three two-week sampling periods (top, middle, bottom panels, respectively), and day (left) and night (right).


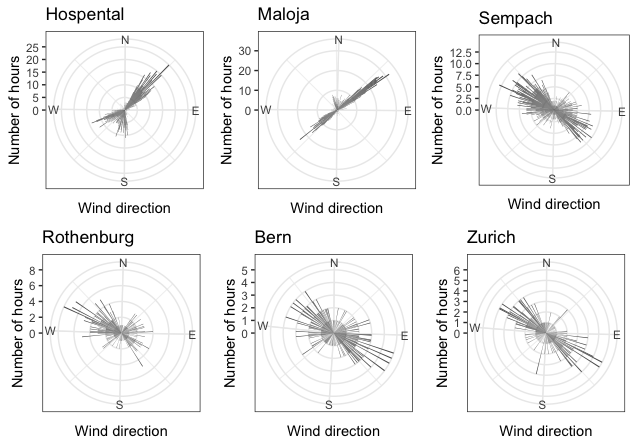


**Supplementary figure 3.** Hourly means of modelled wind directions at the radar sites at a height of 150 m above ground. Similar to wind speed, wind direction (i.e., the direction from which the wind was coming) was extracted from the COSMO-1 model at a height of 150 m above ground. The COSMO-1 is a version of the COSMO (Consortium for small-scale modelling, [www.cosmo-model.org](http://www.cosmo-model.org)) family of numerical weather prediction models with a grid box size of 1.1 km, and it is operated by MeteoSwiss ([www.metoswiss.admin.ch](http://www.metoswiss.admin.ch)). Data extracted for Sempach were also used for Rothenburg, Bern and Zurich. Yet, the wind directions for Rothenburg, Bern, and Zurich differ from those of Sempach as timespans differ.


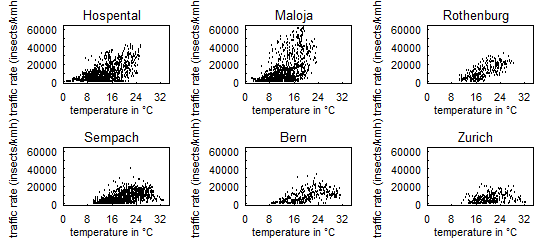


**Supplementary figure 4.** Hourly traffic rate against temperature at the different locations. The slightly positive correlation between temperature and hourly traffic rates seen in the raw data was not found in our multi-variate model (Supplementary table 4). Thus, other variables in the model such as time of the day, air pressure and wind speed may partially explain the slightly positive correlations between temperature and hourly traffic rate.

**
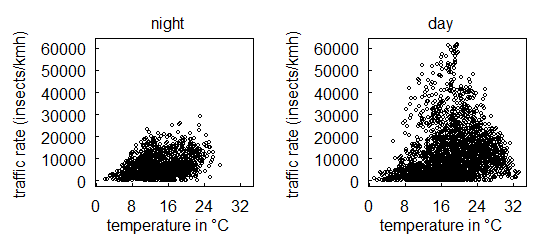
**

**Supplementary figure 5.** Hourly traffic rate against temperature for during the night-time (before dusk, after dawn), during the daytime, respectively.
